# Mature dendritic cells suppress tenocyte and fibroblast function via type I interferon signaling

**DOI:** 10.3389/fimmu.2026.1933065

**Published:** 2026-10-14

**Authors:** Veronica Pinnarò, Sonia V. Rodriguez Monje, Sarojinidevi Künig, Nico Lamot, Adelheid Elbe-Bürger, Maria Camilla Ciardulli, Giovanna Della Porta, Johannes Stöckl

**Affiliations:** 1 Center for Pathophysiology, Infectiology and Immunology, Institute of Immunology, Medical University of Vienna, Vienna, Austria; 2 Department of Dermatology, Medical University of Vienna, Vienna, Austria; 3 Department of Medicine, Surgery and Dentistry, University of Salerno, Salerno, Italy

**Keywords:** dendritic cells, fibroblast, tendinopathies, tenocytes, type I interferon (IFN)

## Abstract

Dendritic cells (DCs) play a critical role in modulating immune responses and tissue homeostasis. While immature DCs have been shown to enhance cell proliferation, the function of mature DCs (mDCs) in connective tissue biology remains less well understood. Here, we report that mDCs exert a potent inhibitory effect on tenocytes (TCs) and fibroblasts (FBs), suppressing both cell proliferation and type I collagen production. Using *in vitro* co-culture and supernatant transfer experiments, we demonstrate that this suppression is primarily mediated by soluble factors. Further mechanistic analysis revealed that type I interferons (IFN-α, IFN-β, IFN-ω), produced by mDCs upon LPS-induced maturation, are key mediators of these effects. Neutralization of the type I IFN signaling pathway restored both proliferation and collagen synthesis in TCs and FBs, confirming the central role of IFN signaling. Our findings suggest that mDCs may contribute to impaired tissue regeneration in inflammatory tendon pathologies.

## 1 Introduction

Tendons are a specialized connective tissue that connect muscles to bone and enable the body to transmit forces generated by muscle contraction. For this reason, they are often referred to as the “mechanical bridges” of the body. The ability of tendons to withstand and transmit mechanical forces relies on their unique structural organization, which is maintained by tenocytes (TCs), tendon-resident cells, and fibroblast-like cells (FBs). In addition, tendons contain resident immune cells that play a central role in tissue homeostasis and contribute to the development and progression of tendon disorders, including tendinopathy (1–3).

The importance of myeloid cells in tendon repair is well established (4–7). In particular, macrophages are known to be essential for tendon regeneration. Dendritic cells (DCs) are part of the myeloid cell compartment and are professional antigen-presenting cells crucial for initiating and regulating immune responses (8, 9). The presence of DCs in tendons has only recently been described, but their functional role remains poorly understood (5). Importantly, DCs have been reported to contribute to tissue repair and wound healing in several tissues. We have previously demonstrated that co-culturing TCs with immature, monocyte-derived DCs treated with OxPAPC (Ox-DCs) enhances the proliferation of tenocytes, particularly those derived from diseased tendons (10). Using transcriptional profiling of Ox-DCs, we identified amphiregulin (AREG), a ligand for EGFR, as a potential mediator of this proliferation-enhancing effect, which we confirmed using recombinant AREG. Of note, diseased tenocytes were found to express higher levels of EGFR compared to tenocytes isolated from healthy donors and showed a stronger proliferative response upon co-culture with Ox-DCs as well as AREG treatment. In summary, we identified a DC-TC crosstalk leading to increased tenocyte proliferation and potentially supporting tendon regeneration (10).

The function of DCs depends on their maturation status. Immature DCs (iDCs) have a high capacity to take up antigens but a relatively poor capacity to activate T cells. In addition, iDC are often considered to promote tissue repair by enhancing FB and stromal cell activity. In contrast, mature DCs (mDCs) have a poor antigen-uptake capacity but represent the most potent inducers of T cell proliferation (11, 12). Since mDCs produce high amounts of several inflammatory cytokines and factors, they are generally associated with pro-inflammatory functions in tissue remodeling and inflammatory responses. The role of mDCs in the context of tendon biology and extracellular matrix (ECM) regulation remains poorly defined (13, 14). Given the critical role of TCs and FBs in ECM synthesis and tendon healing, we aimed to investigate whether mDCs modulate these processes.

Our findings indicated opposing effects of DC subsets on stromal cell behavior, with mDCs reducing the proliferation of TCs and FBs, whereas iDCs promoted their proliferative activity. These observations suggest that distinct DC maturation states may differentially regulate tendon-associated stromal cells and prompted further investigation into molecular mechanisms underlying the inhibitory effects mediated by mDCs. Among the factors potentially involved, we focused on type I interferons (IFNs), a group of cytokines abundantly produced by mDCs and well known for their antiproliferative and immunomodulatory properties.

## 2 Results

### 2.1 mDCs inhibit the proliferation and type I collagen production in hTCs, dTCs, and FBs

We have recently demonstrated that untreated iDCs show a trend toward increased proliferation of the tested cells, but this effect was not significant. However, upon stimulation of iDCs with oxidized phospholipids, the proliferation of TCs was enhanced (10). To study the impact of mDCs on TCs, we cultured primary human TCs obtained from the tendon of a healthy donor (hTC) and a diseased donor (dTC) and compared their proliferative response and collagen production with those of primary human FBs isolated from skin.

Results presented in **Supplementary Figure 1** demonstrate that both types of TCs showed a lower proliferative capacity compared to FBs. In line with this, TCs produced significantly lower amounts of type I collagen compared to FBs (**Supplementary Figure 2**). In contrast, neither iDCs nor macrophages altered the proliferation or collagen production of hTCs, dTCs, or FBs. Co-culture of hTCs, dTCs, and FBs with mDCs generated by LPS stimulation significantly inhibited the proliferative response of the cells and reduced their collagen production (**Figures 1A, B**). DCs (iDCS, mDCs) and macrophages (M1, M2) themselves did not proliferate or produce collagen (**Supplementary Figures 1**, **2**).

**Figure 1:**
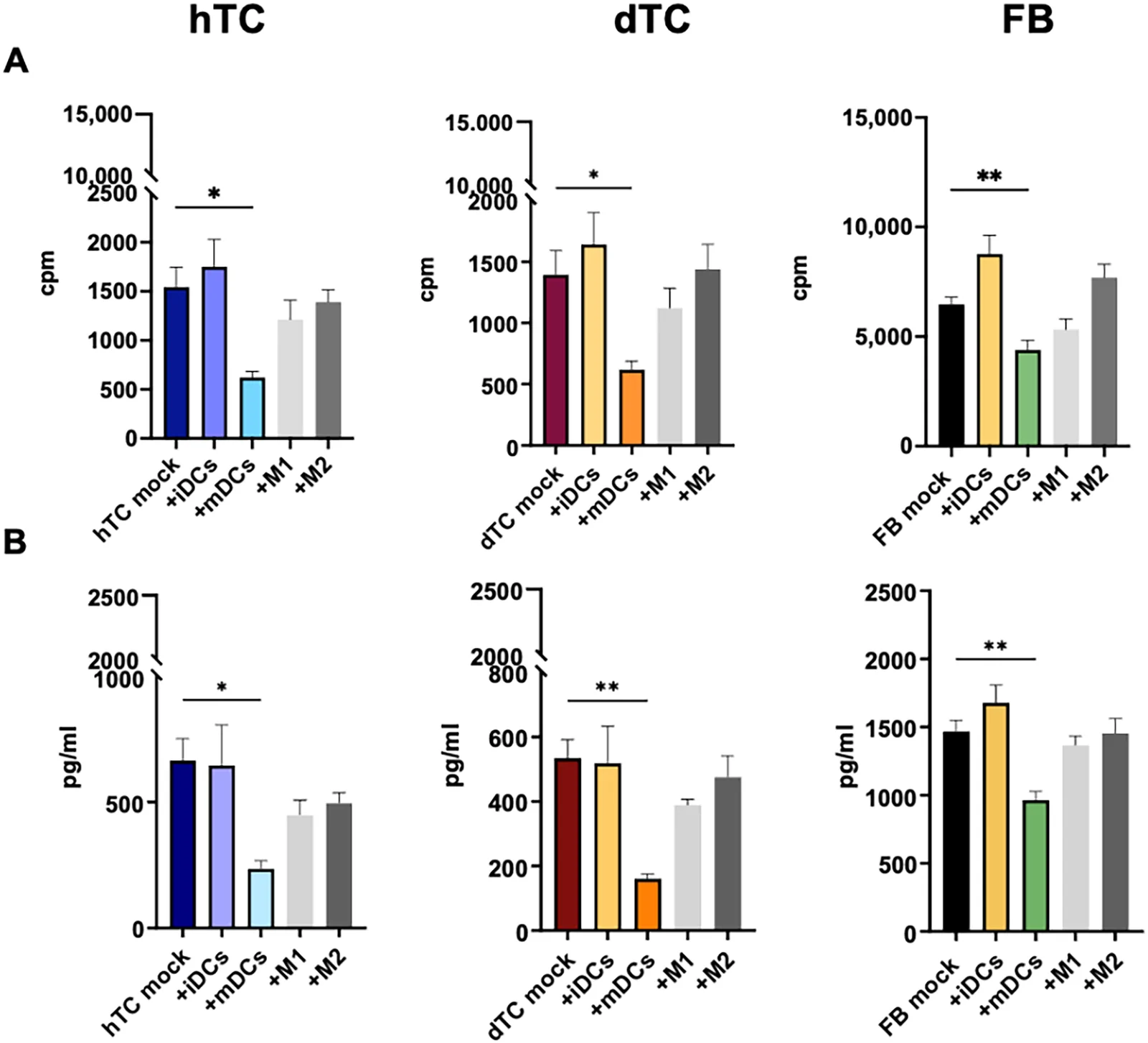
mDCs inhibit proliferation and type I collagen production in tenocytes and fibroblasts. **(A)** hTCs, dTCs, and FBs were co-cultured with mDCs, and proliferation was assessed on day 2 by [^3^H] ‑thymidine incorporation. Data are expressed as counts per minute (cpm). **(B)** Type I collagen production was assessed after 2 days of co-culture by enzyme-linked immunosorbent assay (ELISA) using a COL1A1 antibody. Values are presented as mean ± SEM from four independent experiments. Statistical analysis was performed using a one-way ANOVA and Dunnett’s multiple comparisons test. Only significant differences are indicated. *p <0.05; **p < 0.01.

### 2.2 Impure LPS, but not ultrapure LPS, induces the inhibitory capacity of mDCs

Stimulation of DCs with LPS is a classical treatment to obtain mDCs. Yet impure LPS is not only a trigger for TLR4 but also for TLR2, possibly other pattern recognition receptors (PRRs), since it contains lipoproteins and lipoteichoic acids. In addition, trace amounts of nucleic acids found in impure LPS can activate TLR3/7/8 (15, 16). To determine whether TLR4 activation alone is sufficient to induce the inhibitory capacity of mDCs, we stimulated mDCs with ultrapure LPS and compared the effects with those induced by impure LPS. Data presented in **Figure 2** demonstrate that only mDCs stimulated with impure LPS, but not those stimulated with ultrapure LPS, were able to block the proliferation (**Figure 2A**) and collagen-production (**Figure 2B**) of co-cultured FBs. Treatment of hTCs, dTCs, and FBs with impure LPS alone had no impact on the proliferation or collagen production of the cells (**Supplementary Figure 3**).

**Figure 2:**
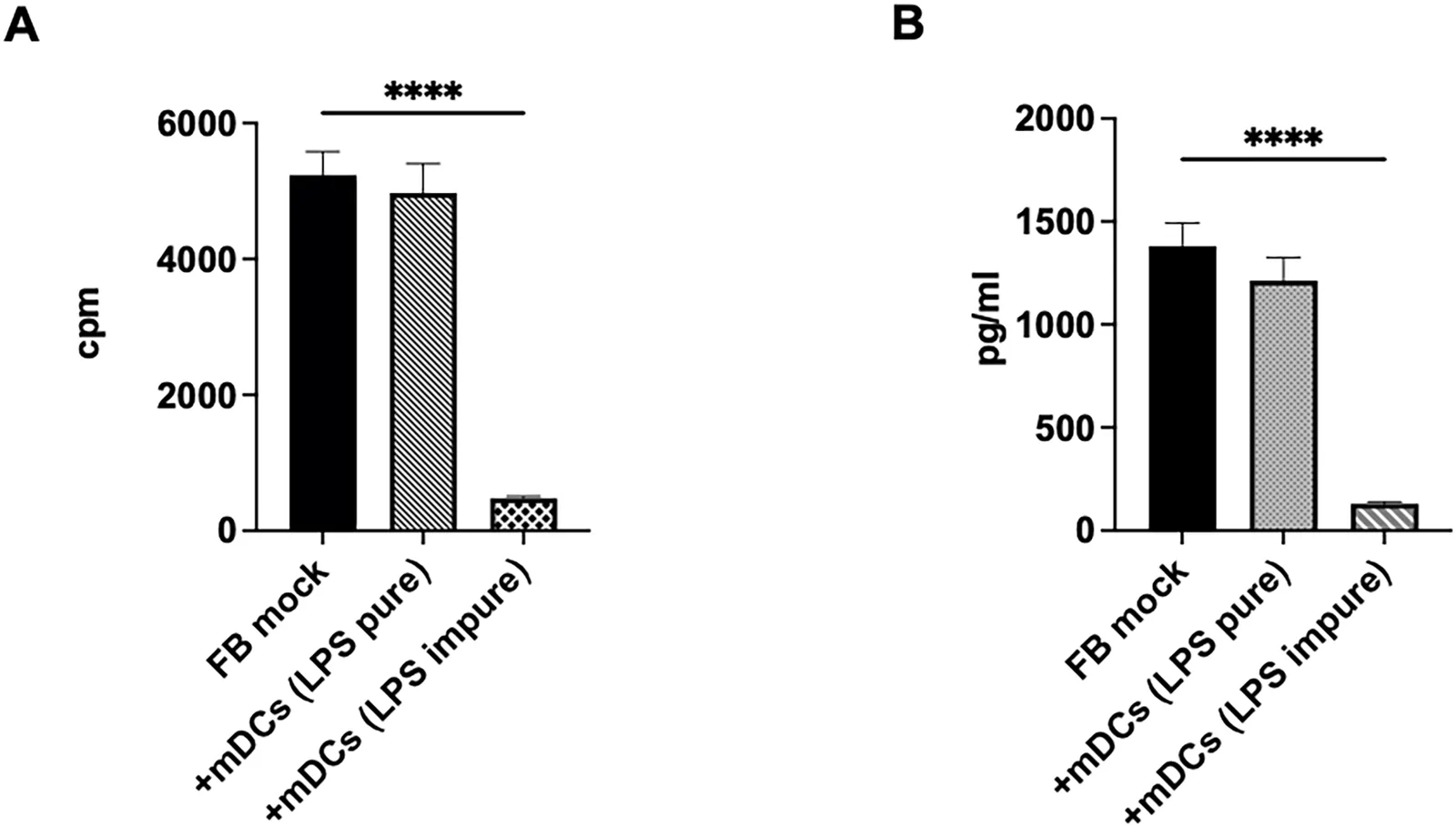
Dendritic cells matured with impure LPS inhibit fibroblast proliferation and type I collagen production. **(A)** Fibroblasts (FBs) were harvested and seeded, and after 48 hours of incubation, [^3^H]-thymidine was added. Proliferation was assessed the following day by measuring thymidine incorporation, expressed as counts per minute (cpm). **(B)** FBs were seeded, and after treatment, the cells were removed, and collagen production was quantified using a COL1A1 antibody. Optical density was measured and converted to pg/ml. Data are shown as mean ± SEM from 3 independent experiments. Statistical analysis was performed using one-way ANOVA followed by Dunnett’s multiple comparisons test. ****p < 0.0001. Only significant differences are shown.

### 2.3 mDC-derived supernatants inhibit proliferation and collagen type I production in TCs and FBs

Induction of DC maturation by stimulation with LPS is accompanied by extensive molecular reprogramming, including the production and release of multiple soluble factors and cytokines. To determine whether the inhibitory effects of mDCs on TCs and FBs depend on direct cell-cell contact or are mediated by soluble factors, we next tested supernatants (SNs) from mDCs on our cells. mDC-derived SNs inhibited the proliferation (**Figure 3A**) and collagen production (**Figure 3B**) of hTCs, dTCs, and FBs. In comparison to the strong and significant inhibition obtained with SNs from mDCs, we did not observe such an effect with SNs from iDCs or macrophages (M1, M2). Treatment with iDC- or mDC-derived SNs did not affect the viability of TCs or FBs (**Supplementary Figure 4**).

**Figure 3:**
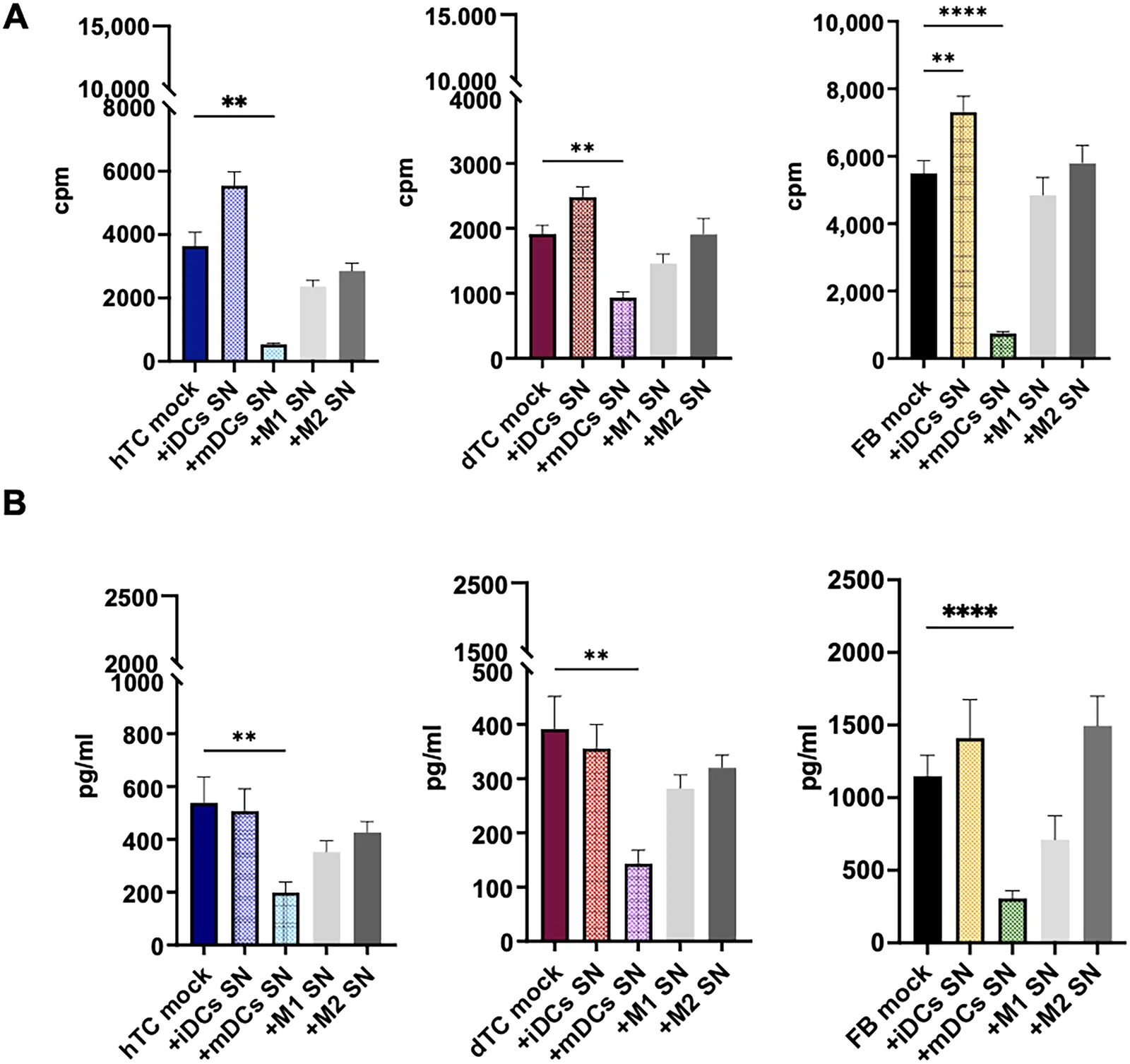
mDCs-derived supernatant negatively regulates proliferation and type I collagen production in tenocytes and fibroblasts. **(A)** Proliferation rates of healthy tenocytes (hTCs), diseased tenocytes (dTCs), and fibroblasts (FBs) after treatment with immature dendritic cell (iDC)-derived-supernatant (SN) or matured-DC-derived SN, assessed by thymidine incorporation and expressed as count per minute (cpm) after 2 days of treatment. **(B)** Type I collagen production was measured by enzyme-linked immunosorbent assay (ELISA). Data are presented as mean ± SEM from four independent experiments. Statistical analysis was performed using one-way ANOVA, followed by Dunnett’s multiple comparisons test. **p < 0.01, ****p < 0.0001. Only significant differences are shown.

### 2.4 Type I interferon signaling inhibits TC and FB proliferation

To identify soluble factors responsible for the inhibitory effect of mDCs, we tested the functional effects of a set of prominent cytokines on FBs. Neither TNFα, IL-10, IL-9, IL-4, IL-6, nor IL-1 influenced FB proliferation and collagen production (**Supplementary Figure 5**).

Given the well-described antiproliferative properties of type I interferons and their known production by activated/mature DCs, we next investigated whether type I IFN signaling may contribute to the inhibitory effects of mDC-derived soluble factors on TCs and FBs. As shown in **Figure 4A**, all three cell types exhibited upregulation of Myxovirus resistance protein A (MxA), an intracellular protein specifically induced by IFN-α/β stimulation (17, 18). Interestingly, the response was significantly higher in TCs than in FBs. As shown in **Figure 4B**, all three cell types expressed IFNAR, which mediates signaling in response to type I IFNs such as IFN-α and IFN-β. However, FBs expressed significantly higher levels of the IFN receptor. Thus, despite lower IFN receptor expression, TCs appear to be more sensitive to type I IFN stimulation than FBs.

**Figure 4:**
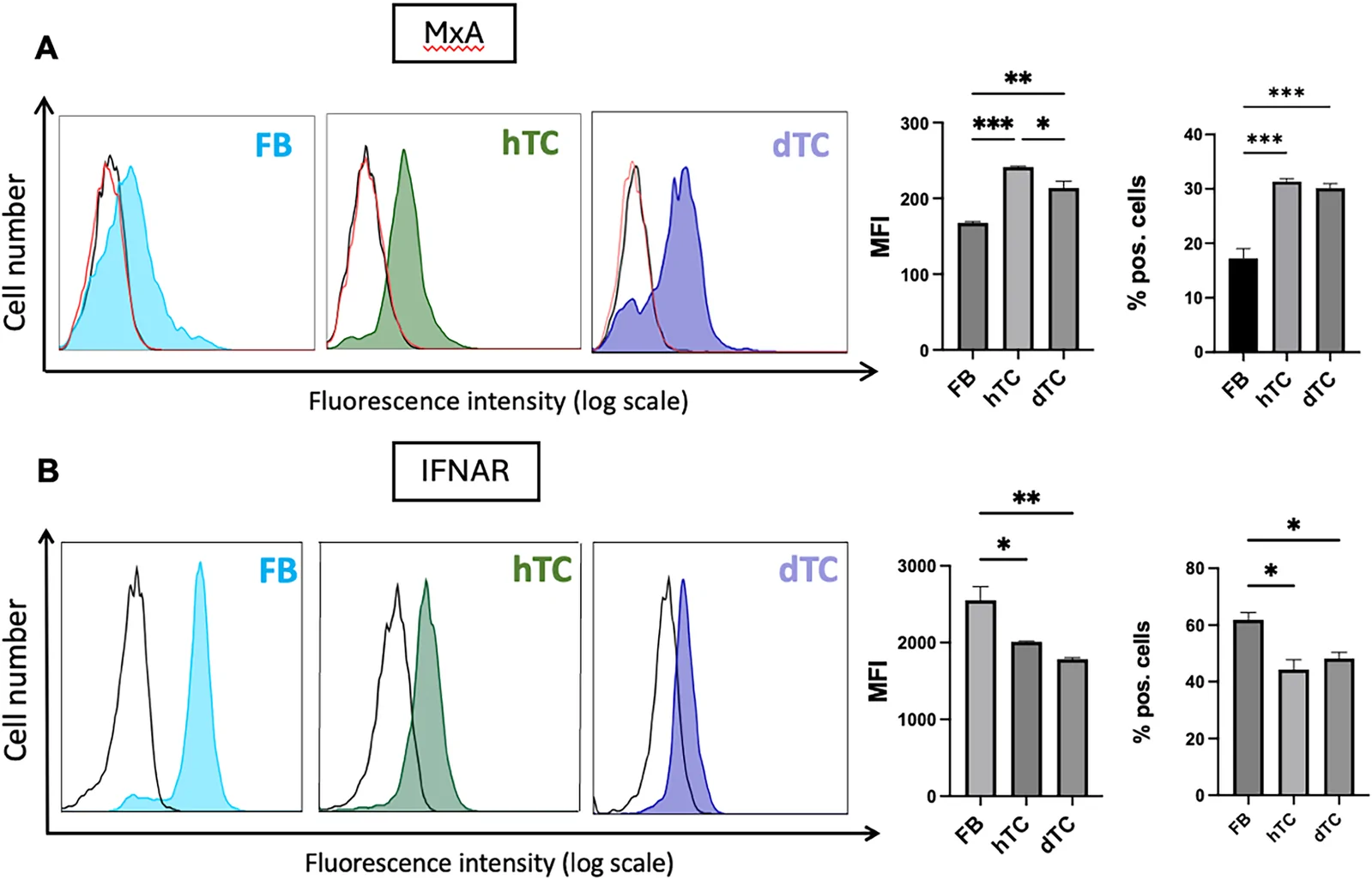
Type I interferon pathway activation upon treatment with mature dendritic cell-derived supernatant. **(A)** Fibroblasts (FBs; light blue), healthy tenocytes (hTCs; dark green), and diseased tenocytes (dTCs; purple) were treated with mature dendritic cell-derived supernatants for 48h. Intracellular MxA expression was analyzed by flow cytometry. Representative histograms show MxA fluorescence intensity in untreated cells (red line) compared with unstained controls (black lines). Quantification is shown as mean fluorescence intensity (MFI) and percentage of MxA-positive cells. **(B)** Surface expression of IFNAR, the receptor complex mediating signaling by type I IFNs, including IFN-α and IFN-β. Statistical analysis was performed using one-way ANOVA, and only significant differences are indicated: *p < 0.05, **p < 0.01, ***p < 0.001. Bar graphs show mean ± SEM from *n* = 3 biological replicates.

Next, we studied whether blockade of type I IFN signaling could reverse the inhibitory effect of mDC-derived SNs in hTCs, dTCs, and FBs. To this end, we used a comprehensive approach targeting simultaneously the receptor (IFNAR) and the associated signaling proteins, IFN-α, IFN-β. This approach was based on previous studies that used the same combination of neutralizing antibodies to block type I IFN signaling (19). Results presented in **Figure 5** demonstrate that blocking the type I IFN signaling significantly reduced the inhibitory effect of SNs produced by mDCs. We also tested the antibodies against type I IFN receptor (AB1), IFN-α2 (AB2), and IFN-β (AB3) separately and in combination. Blocking the IFN receptor alone (AB1) was enough to restore proliferation and collagen production in hTCs, dTCs, and FBs, similar to the combined AB1–3 treatment, while AB2, AB3, or their combination (AB2-3) had little to no effect (**Supplementary Figure 6**). It is well established that DCs can produce type I IFNs upon stimulation with impure LPS (20, 21). To confirm that this was also the case in our system, we measured type I IFNs at the protein level in the supernatants of our mDCs (**Supplementary Figure 7**).

**Figure 5:**
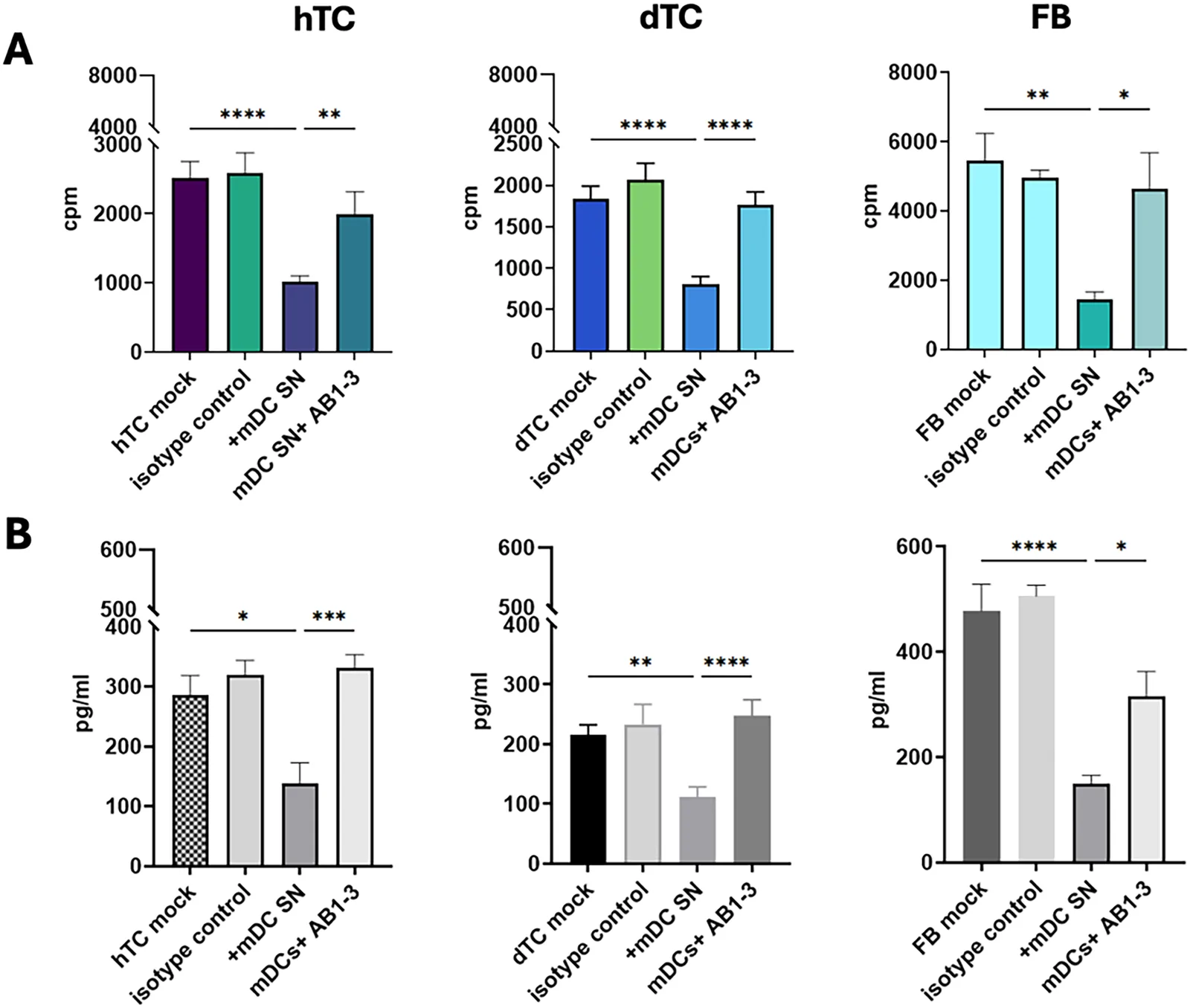
Blocking of type I IFN signaling reverses the inhibitory effects of mature dendritic cell-derived supernatants. **(A)** Proliferation assay of tenocytes (TCs) and fibroblasts (FBs) treated with supernatant (SN) from LPS-stimulated dendritic cells (mDCs) or left untreated (mock). mDC-derived SNs were supplemented with a cocktail of blocking antibodies targeting the type I IFN receptor IFNAR, IFN-α2, and IFN-β, labelled as AB 1-3. Proliferation was measured by thymidine incorporation after 48 hours, with results expressed as counts per minute (cpm). **(B)** Collagen production was evaluated by enzyme-linked immunosorbent assay (ELISA). Cells were cultured for 2 days, after which SNs were collected, and type I collagen (COL1A1) levels were measured. Data represent the mean ± SEM from 3 independent experiments. Statistical analysis was conducted using a one-way ANOVA, and only significant differences are indicated: *p < 0.05, **p < 0.01, ***p < 0.001, ****p < 0.0001.

To further validate the previous findings, we tested the effects of recombinant IFN-α, IFN-β and IFN-ω and their combination. hTCs, dTCs, and FBs were treated with the recombinant factors for 48 hours, followed by assessment of cell proliferation and collagen production. As shown in **Figure 6**, all tested type I IFN subtypes significantly inhibited both proliferation and collagen production in TCs and FBs, without affecting cell viability (**Supplementary Figure 4**). These data support the conclusion that type I IFNs are key mediators of the inhibitory effects observed in our cell culture system.

**Figure 6:**
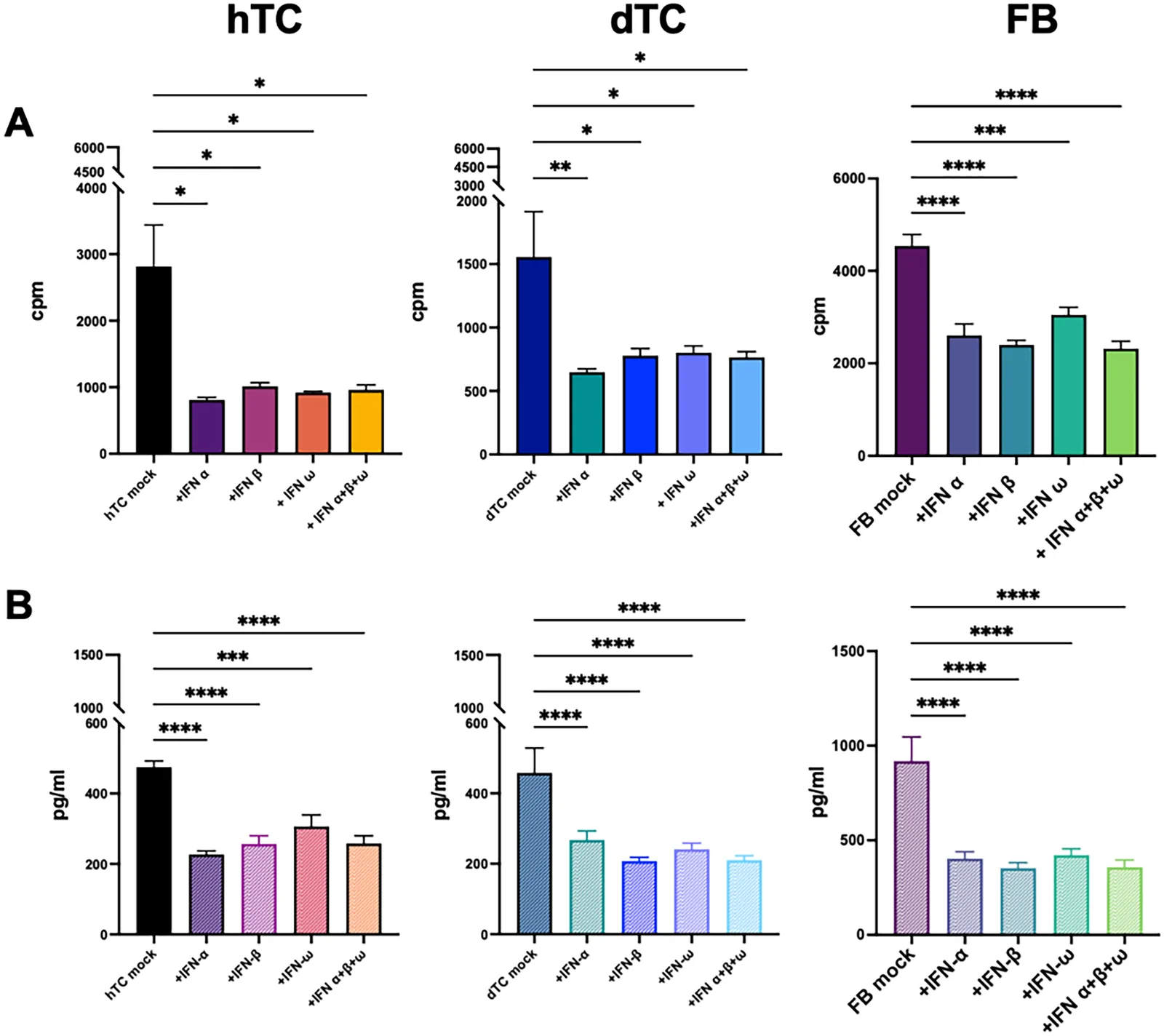
Recombinant type I interferons inhibit proliferation and collagen production. **(A)** Proliferation activity of healthy tenocytes (hTC), diseased tenocytes (dTC), and fibroblasts (FB) in response to treatment with interferon (IFN)-α, IFN-β, IFN-ω, or a combination of all three IFNs. Mock-treated cells served as controls. **(B)** Type I collagen production (pg/mL) in hTCs, dTCs, and FBs following IFN treatment, as measured by ELISA. Data are expressed as mean ± SEM from three independent experiments. Statistical analysis was conducted using one-way ANOVA followed by Dunnett’s multiple comparisons test. Significant differences between groups are indicated as follows: *p<0.05; **p<0.01; ***p<0.001; ****p<0.0001.

## 3 Discussion

The present study identifies mDCs as potent regulators of stromal cell function, demonstrating their ability to suppress both proliferation and collagen type I production in TCs and FBs. In contrast, iDCs promoted proliferative activity, highlighting a maturation-dependent functional switch. Mechanistically, our data establish type I IFNs, such as IFN-α, IFN-β, and IFN-ω, as principal mediators of these inhibitory effects.

A key aspect of these findings is the distinction between DCs and macrophages, which are often studied in parallel in the context of tissue repair. Classically activated M1 macrophages, frequently generated using LPS stimulation, predominantly produce pro-inflammatory cytokines such as TNF-α and IL-1β (22). Although these mediators can modulate FB behavior, they do not typically induce a sustained type I IFN response. In contrast, activated/matured DCs are capable of inducing type I IFNs, particularly following engagement of nucleic acid-sensing PRRs (23, 24). This response is most prominently associated with viruses (23, 25–27). Yet, the simultaneous activation of multiple PPRs, such as TLRs, by exogenous ligands like impure LPS or endogenous alarmins released during tissue damage may also promote type I IFN production by DCs (28–30). The induction of type I IFNs is closely linked to activation of Toll-like receptor (TLR) signaling, particularly via endosomal receptors such as TLR3, TLR7, and TLR9, as well as cytosolic nucleic acid sensors (31). As the contribution of these pathways to tendon inflammation remains poorly explored, future studies using other TLR agonists, such as polyinosinic:polycytidylic acid (poly(I:C)), Pam3CSK4, or resiquimod, could help clarify their role in DC-mediated type I IFN responses in the context of tendon injury. This requirement for complex PRR engagement suggests that the observed mDC phenotype may resemble immune responses triggered by viral infection or endogenous danger-associated molecular patterns (DAMPs). Such a context may be highly relevant in tendon injury, where tissue damage can lead to the release of nucleic acids and alarmins such as S100 proteins, which may contribute to IFN-inducing inflammatory responses. Interestingly, tendon-related symptoms and tendinopathies have been reported in association with SARS-CoV-2 infection and, in rare cases, following COVID-19 vaccination, although a causal relationship remains to be fully established (32, 33).

The interplay between primary human DCs and stromal cells, i.e., fibroblasts and tenocytes, is still incompletely understood. Moreover, the contribution of different DC subsets to tendon homeostasis, injury, and repair has received little attention. This includes plasmacytoid DCs (pDCs), which are major producers of type I IFNs and have recently been identified in ruptured human tendons (34), but their role and function remain largely unexplored. Further investigation of these cells will be important to better define how they contribute to the local immune response and influence tendon healing and repair.

We have analyzed this interaction in a basic *in vitro* system. It is, of course, not possible to directly translate this information into the biological relevance of these findings for the *in vivo* situation in homeostatic or diseased tissues. Yet, there are clinical situations that fit and support our *in vitro* results. For instance, autoimmune diseases are characterized by chronic cytokine dysregulation and, in particular, activation of type I IFN pathways. Systemic lupus erythematosus (SLE) exhibits a well-described type I IFN signature, while type I IFN pathway activation has also been reported in subsets of patients with rheumatoid arthritis (35–37). Both diseases are associated with musculoskeletal manifestations, including tendon-related involvement. Our results may provide a potential mechanistic link between type I IFN exposure and impaired tendon homeostasis, suggesting that type I IFNs may contribute to reduced tenocyte proliferation and extracellular matrix (ECM) synthesis.

At the same time, an important consideration is the translational gap between *in vitro* models and human disease. Factors such as tissue architecture, immune cell composition, local cytokine gradients, and mechanical loading may influence the extent to which these findings apply *in vivo*. Human tendons, in particular, exhibit limited regenerative potential and are prone to chronic degeneration (38–40), which may render them especially susceptible to the inhibitory effects of sustained IFN signaling. Therefore, while our *in vitro* data provide clear mechanistic insights, further validation in human tissue and *in vivo* models is required.

From a physiological perspective, type I IFN-mediated suppression of stromal cell activity may represent a protective mechanism aimed at limiting excessive tissue remodeling. However, in the context of chronic inflammation or repetitive injury, this response may become maladaptive, leading to impaired healing and ECM dysregulation (13, 41). Type I IFNs are known to exert anti-proliferative effects on many cell types, including tumor cells, as well as stromal cells such as FBs. Moreover, treatment of human FBs with type I IFNs has been shown to reduce collagen type I production (42), which is in line with the findings of the present study.

A limitation of this study is that healthy and diseased tenocytes were each obtained from a single donor, due to the limited availability of tendon-derived material. While dendritic cells and fibroblasts were derived from multiple independent donors across experiments, future studies including tenocytes from additional donors will be needed to confirm the biological reproducibility and significance of these findings.

In conclusion, this study identifies type I IFNs as central negative regulators of stromal cell function and highlights mDCs as key cellular sources of these signals in the context of tissue injury. By linking immune activation to impaired ECM homeostasis, our findings provide a conceptual framework for understanding tendon pathology as an immunologically modulated process. Targeting type I IFN signaling may therefore represent a promising strategy to support tissue regeneration and improve outcomes in degenerative tendon diseases in the future.

## 4 Materials and methods

### 4.1 Isolation of peripheral blood mononuclear cells and generation of moDCs

Buffy coats from four healthy donors were obtained from the Austrian Red Cross (Vienna, Austria). Peripheral blood mononuclear cells (PBMCs) were isolated via standard density gradient centrifugation using Ficoll-Paque™ Plus (GE Healthcare, UK). Monocytes were purified from PBMCs using the MACS system (Miltenyi Biotec, Germany) as described by (15). CD14^+^ monocytes were cultured for 6 days in RPMI 1640 culture medium (Sigma-Aldrich, St. Louis, USA, cat. Number R0883) supplemented with 2 mM L-glutamine (Gibco by Thermo Fisher Scientific, Paisley UK, cat. Number 11539876), 100 μg/mL streptomycin and 100 U/mL penicillin (PAA Laboratories, Pasching, Austria) and 10% heat-inactivated foetal calf serum (Gibco by Thermo Fisher Scientific, UK, cat. Number 10270-106). To generate monocyte-derived dendritic cells (MoDCs), CD14^+^ monocytes were cultured with recombinant human GM-CSF (50 ng/mL; kindly provided by Novo Nordisk A/S, Bagsværd, Denmark) and IL-4 (200 U/mL; kindly provided by Novo Nordisk A/S, Bagsværd, Denmark) for 5 days. To induce maturation into mDCs, cells were stimulated on day 5 with impure or ultrapure LPS (2 µg/mL; Sigma-Aldrich, St. Louis, USA) for 2 days.

Primary human monocytes were differentiated into macrophages by culturing them with 50 ng/mL M-CSF for 5 days. Macrophages were then polarized toward the M1 phenotype by treatment with 100 ng/mL LPS and 25 ng/mL IFN-γ for an additional 2 days. For M2 polarization, macrophages were instead treated with 25 ng/mL IL-4 for an additional 2 days. M-CSF and IFN-γ were kindly provided by the Novartis Research Institute (Vienna, Austria).

### 4.2 Tenocytes and fibroblasts

TCs were isolated according to a previously described protocol (43) and cultured in αMEM medium supplemented with 10% fetal bovine serum (FBS; Gibco Ltd., Paisley, UK), 1% penicillin/streptomycin (P/S; PAA Laboratories, Pasching, Austria), and 0.5% L-glutamine (Gibco Ltd., Paisley, UK). Healthy tenocytes (hTCs) were obtained from excess semitendinosus tendon tissue collected during autologous anterior cruciate ligament reconstruction. Diseased tenocytes (dTCs) were isolated from patients undergoing surgery for tendinopathic injuries of the Achilles tendon. All tendon samples were collected after written informed consent, according to protocols approved by the Institutional Review Board of San Giovanni di Dio e Ruggi D’Aragona Hospital, Salerno, Italy (Review Board prot./SCCE n. 151, approved on 29 October 2020). Human dermal fibroblasts (FBs) were used as an astromal cell model because of their structural similarity to TCs. Four different donors were used for the experiments. Skin samples from anonymous healthy female donors were obtained from the abdomen, breast, or back during plastic surgery procedures. The study was approved by the Ethics Committee of the Medical University of Vienna (ECS 1969/2021) and conducted in accordance with the Declaration of Helsinki. Written informed consent was obtained from all participants. FBs were isolated as previously described (44, 45) and cultured in a T-75 flask (Corning, Costar) in Dulbecco’s Modified Eagle Medium (DMEM) supplemented with 10% FBS and 1% P/S.

### 4.3 Cell proliferation assay

TCs or FBs were seeded in 96-well plates (Corning Costar, Arizona, USA) at a density of 5x10^3^ cells per well. Cells were cultured either in co-culture with DCs or treated with the respective stimuli, including LPS, DC-derived supernatants, cytokines, or IFNs, for 48 h. The supernatants were generated during the differentiation of DCs/macrophages in culture flasks. At the end of the 7-day differentiation period, the supernatants were collected for each experiment and either used immediately or stored at −20 °C until use. For the treatment experiments, 5×10^3^ fibroblasts or tenocytes per well were seeded in 96-well flat-bottom plates (Corning Costar, Arizona, USA) and treated with 50 µL SN for 2 days. The supernatants were used without prior dilution.

Subsequently, cells were labelled with 1.85/MBq/well of [methyl-^3^H]-thymidine (Perkin Elmer, Waltham, USA) and cultured for an additional 18 h at 37 °C before harvesting. Cells were harvested using a Unifilter-96 Cell Harvester SS (Perkin Elmer, Waltham, USA), and the filter plate was dried for 3 hours in a dry incubator. Scintillation cocktail (25 µl/well; Perkin Elmer, Waltham, USA) was then added to each well, and the thymidine incorporation was measured by a MicroBeta Microplate Counter (Perkin Elmer, Waltham, USA). Readings are displayed as counts per minute (cpm). All cytokines (TNF-α, IL-10, IL-9, IL-4, IL-6, IL-1α, and IL-1β) were used at a concentration of 1 ng/mL.

### 4.4 Collagen type I production analysis by enzyme-linked immunosorbent assay

Collagen deposition was assessed using a modified version of the Human Pro-Collagen I α1 DuoSet ELISA (R&D Systems), adapted to measure collagen deposited onto the culture plate rather than collagen present free in the culture supernatant, as previously described (10). Briefly, 5x10^3^ cells/well were seeded and incubated for 48 h. Cells were then removed by washing with distilled water. After confirming that no cells remained, the wells were washed with washing buffer consisting of PBS (Corning, USA 21-040-CV) containing 0.05% Tween-20 (Bio-Rad Laboratories, Vienna, Austria). The detection antibody from the human pro-collagen I α1 DuoSet ELISA (R&D Systems, Minneapolis, USA) was added at a final concentration of 50 ng/mL. After incubation for 2 h at room temperature, the wells were washed to remove unbound antibody. Streptavidin-HRP (R&D Systems, Minneapolis, USA) was then added and incubated for 30 min, followed by the addition of TMB-substrate solution prepared by mixing reagents A and B at a 1:1 ratio (R&D Systems). After 30 min incubation in the dark, the stop solution was added. Finally, optical density was measured with a Biosan HiPo MPP-96 microplate reader with QuantAssay software at 450 nm, with wavelength correction at 570 nm. Absorbance values (OD) were converted to concentrations (pg/mL) by interpolation from a standard curve generated using the recombinant protein standard provided in the kit, fitted with a four-parameter logistic (4PL) nonlinear regression model (GraphPad Prism, version 9.0-10.0).

### 4.5 Recombinant IFN treatment and IFN neutralization assays

Cells were treated with recombinant human IFN-α, IFN-β, and IFN-ω, or a combination of all three for 48 h. For neutralization assays, three monoclonal antibodies, referred to as AB1-3, were used to block type I IFN signaling. AB1 was a mouse anti-human IFN α/β receptor chain 2 antibody targeting IFNAR2 (Millipore, MAB1155), used at a concentration of 1 µg/mL to block the type I INF receptor. AB2 was an anti-human IFN-α antibody (eBioscience, BMS160). and AB3 was an anti-human IFN-β antibody (eBioscience, BMS1044), each used at a concentration of 1.33 µg/mL. To evaluate the effect of IFN treatment and IFN neutralization, cell proliferation and collagen type I deposition were assessed after 48 h of incubation at 37 °C in 5% CO_2_. Mouse mAb 1-162 (directed against IL-1RAP) is an IgG1 antibody produced at the Institute of Immunology, Vienna, Austria, and was used as an isotype control (1 µg/mL). All experiments were performed independently three times, with each condition analyzed in triplicate.

### 4.6 Type IFNs measurement

To assess type I IFN production by dendritic cells, immature DCs were stimulated with LPS (1 µg/mL) or left untreated and cultured for 2 days. Cell culture supernatants were then harvested and stored at −20 °C until further analysis. IFN-α, IFN-β, and IFN-ω levels were measured using the MILLIPLEX Map Kit (Merck, Darmstadt, Germany) - Luminex system, as described in the manufacturer’s protocol. All measurements were performed in triplicate.

### 4.7 Flow cytometric analysis of IFNAR surface expression and intracellular MxA expression

For cell surface staining, 5x10^4^ cells were incubated with an unconjugated anti-IFNAR antibody for 30 min at 4 °C. After two washing steps, samples were incubated for an additional 30 min with Alexa Fluor 488 goat anti-mouse IgG secondary antibody (#2379467, 1:100, Invitrogen, Waltham, MA, USA). For intracellular MxA staining, cells were harvested and fixed/permeabilized using the Fix&Perm kit (Invitrogen, Thermo Fisher Scientific, Waltham, MA, USA) according to the manufacturer’s instructions. Briefly, cells were resuspended in 100 µL fixation medium and incubated for 15 min at room temperature. After washing with PBS and centrifugation for 5 min, the cell pellet was resuspended in 100 µL of permeabilization medium containing anti-MxA antibody and incubated for 15 min at room temperature. Following two washes with PBS, Alexa Fluor 488 goat anti-mouse IgG (Invitrogen, Waltham, MA, USA, #2833436) was used as a secondary antibody. For both staining procedures, cells were resuspended in sheath fluid and immediately analyzed using an LSR Fortessa flow cytometer (BD Bioscience, Franklin Lakes, NJ, USA). Data were analysed with FlowJo™ Software (BD Life Sciences, New Jersey, USA).

### 4.8 Statistical analysis

Statistical analyses were conducted using GraphPad Prism Software versions 9.0-10.0 (GraphPad Software, Inc., La Jolla, CA, USA). Comparisons among multiple groups were performed using one-way ANOVA, followed by Dunnett’s multiple comparison *post hoc* test. Data are presented as mean ± standard error of the mean (SEM). p-values below 0.05 were considered statistically significant. Statistical significance is indicated as follows: *p < 0.05, **p < 0.01, ***p < 0.001, and ****p < 0.0001. Flow cytometry data were analyzed using FlowJo™ software version 10.6.1 (Becton Dickinson, Franklin Lakes, NJ, USA).

## Data Availability

The raw data supporting the conclusions of this article will be made available by the authors, without undue reservation.
