# Supplementary material for: Mature dendritic cells suppress tenocyte and fibroblast function via type I interferon signaling
Source: Front Immunol. 2026 Oct 14;17:1933065. doi: 10.3389/fimmu.2026.1933065 (PMC13713368; doi:10.3389/fimmu.2026.1933065)
Supplement: Supplementary file 1 [file DataSheet1.pdf]

Supplementary Figure 1

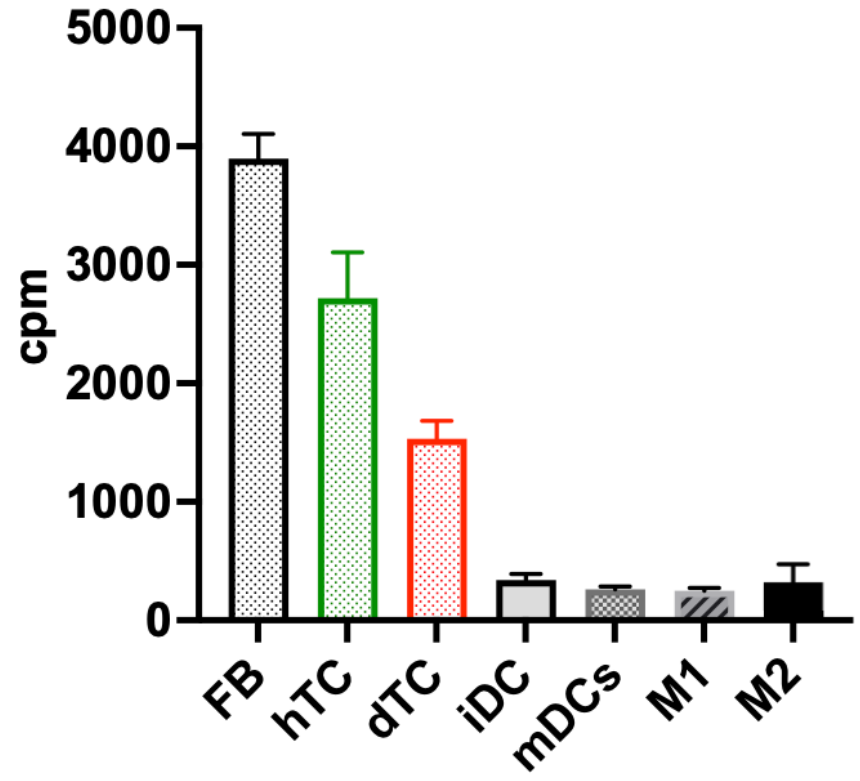

Supplementary Figure 1. Fibroblasts proliferate more than tenocytes and immune cells. Cells were seeded and let proliferate without any additional stimulation for 48 h. Proliferation was then assessed based on incorporated [^{3}H] -thymidine. Data are expressed as counts per minute (cpm). Values are presented as means ± SEM from 3 independent experiments.

Supplementary Figure 2

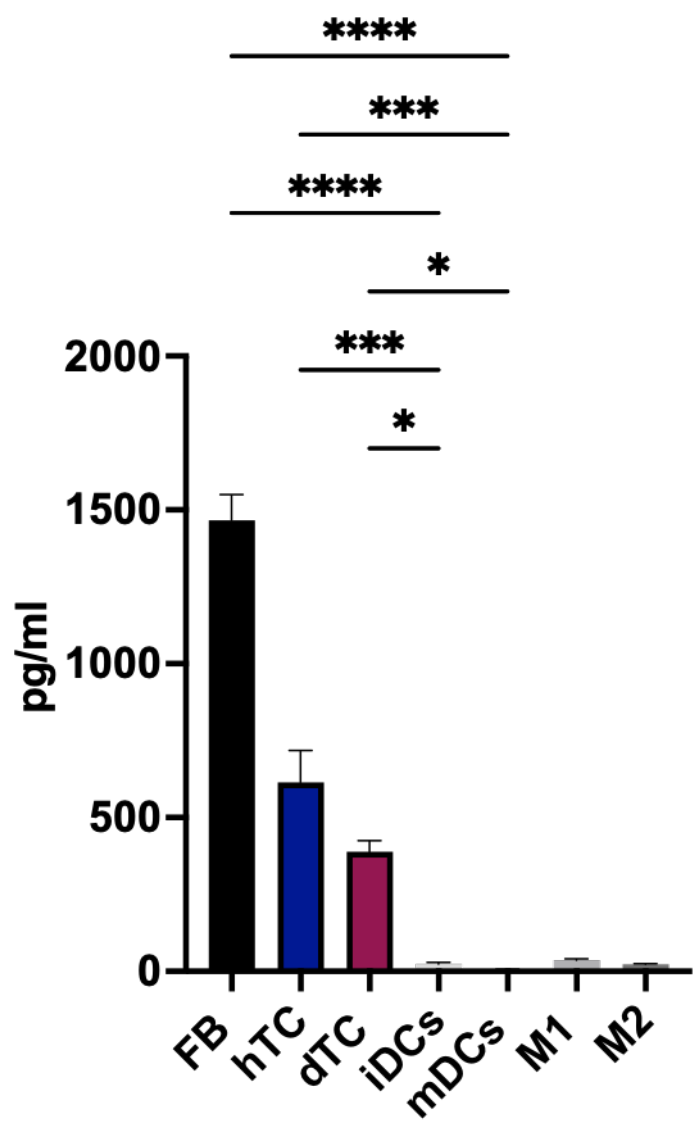

Supplementary Figure 2. Fibroblasts are the predominant source of collagen compared with tenocytes and immune cells. Collagen production was assessed after 2 days of culture and detected using an ELISA assay with a collagen I α 1 antibody. Values are presented as means ± SEM from 3 independent experiments. Statistical analysis was performed using a one-way ANOVA test. Only significant differences are indicated. *$p < 0.05$; *** = $p < 0.001$; ****$p < 0.0001$.

Supplementary Figure 3

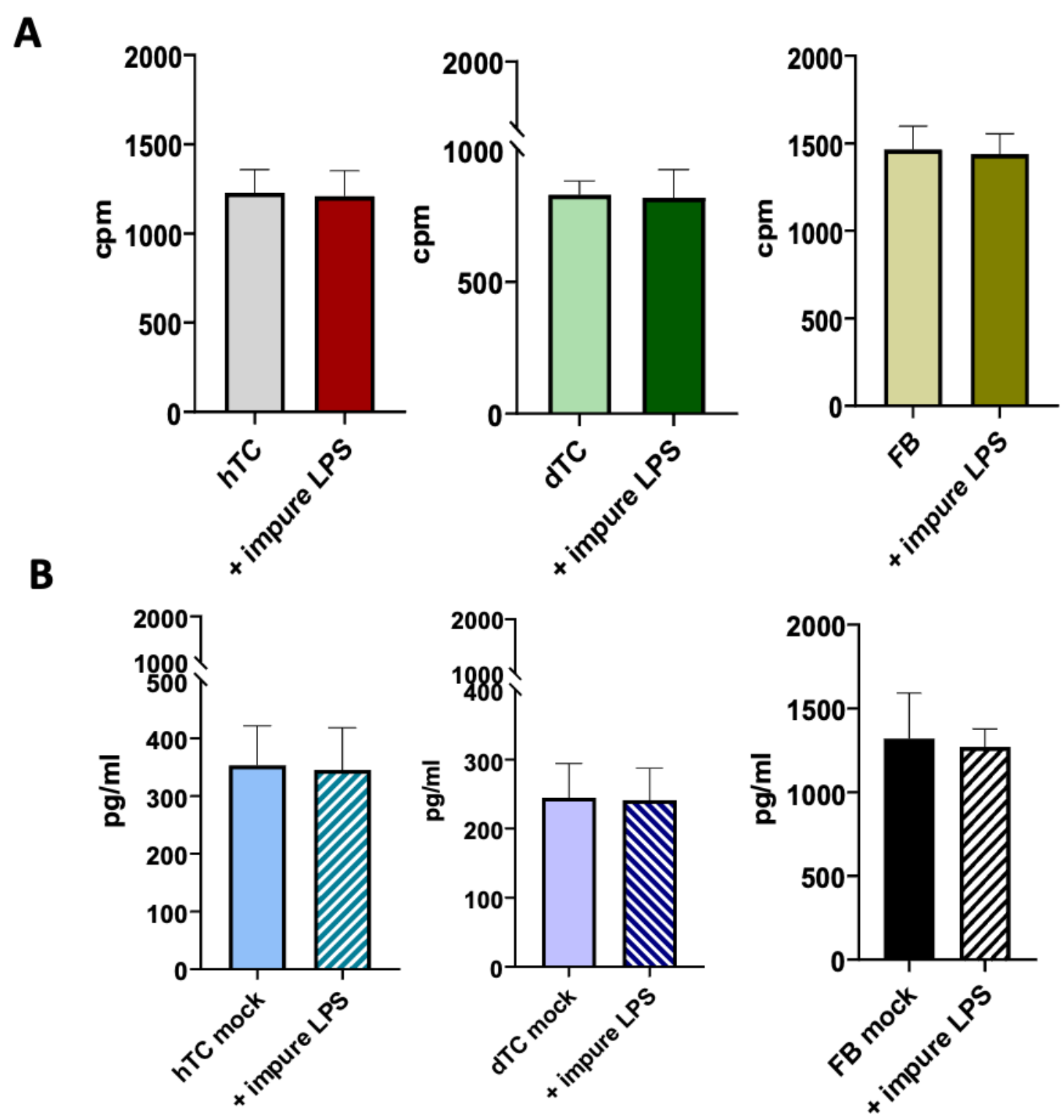

Supplementary Figure 3. Impure LPS alone has no effect on fibroblasts/tenocytes proliferation nor collagen production. (**A**) Cells were seeded in a 96-well plate and treated with impure LPS for 48 hours. Proliferation was assessed with 3H-Thymidine incorporation and expressed as count per minute (cpm). (**B**) Collagen production was measured via ELISA, detecting the produced collagen using a collagen I α 1 antibody. Values are presented as means ± SEM.

Supplementary Figure 4

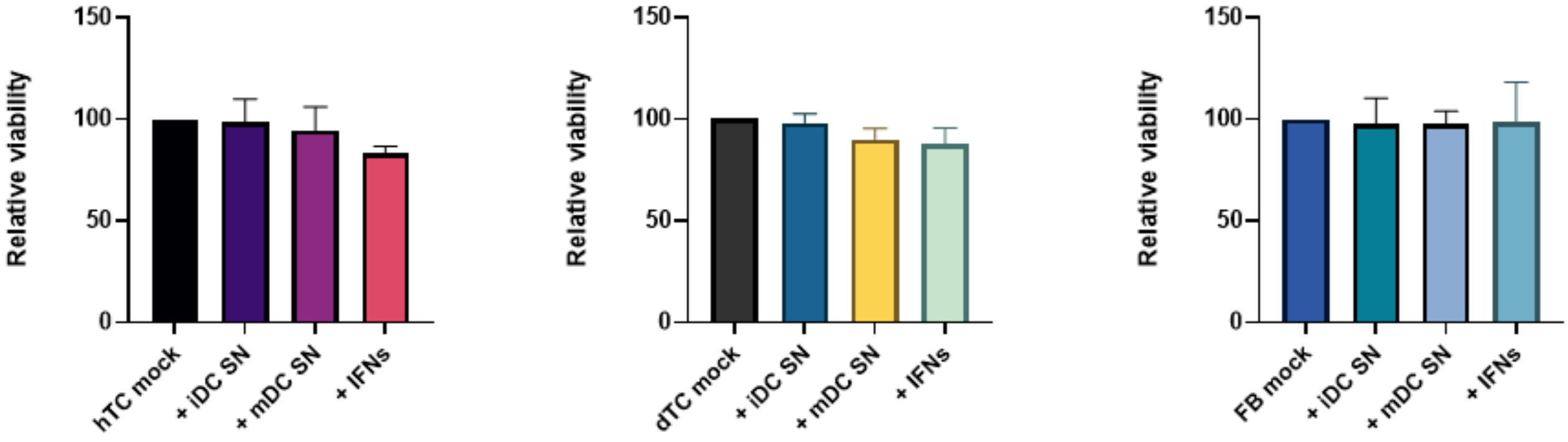

Supplementary Figure 4. Supernatant of mDC or IFNs treatment has an impact on the viability of tenocytes or fibroblasts. Cells were seeded in a 96-well plate and treated for 48 hours with iDCs supernatant, mDCs supernatant, and the combination of IFNs. Viability was assessed using propidium iodide. Error bars: SEM. Values from four independent experiments.

Supplementary Figure 5

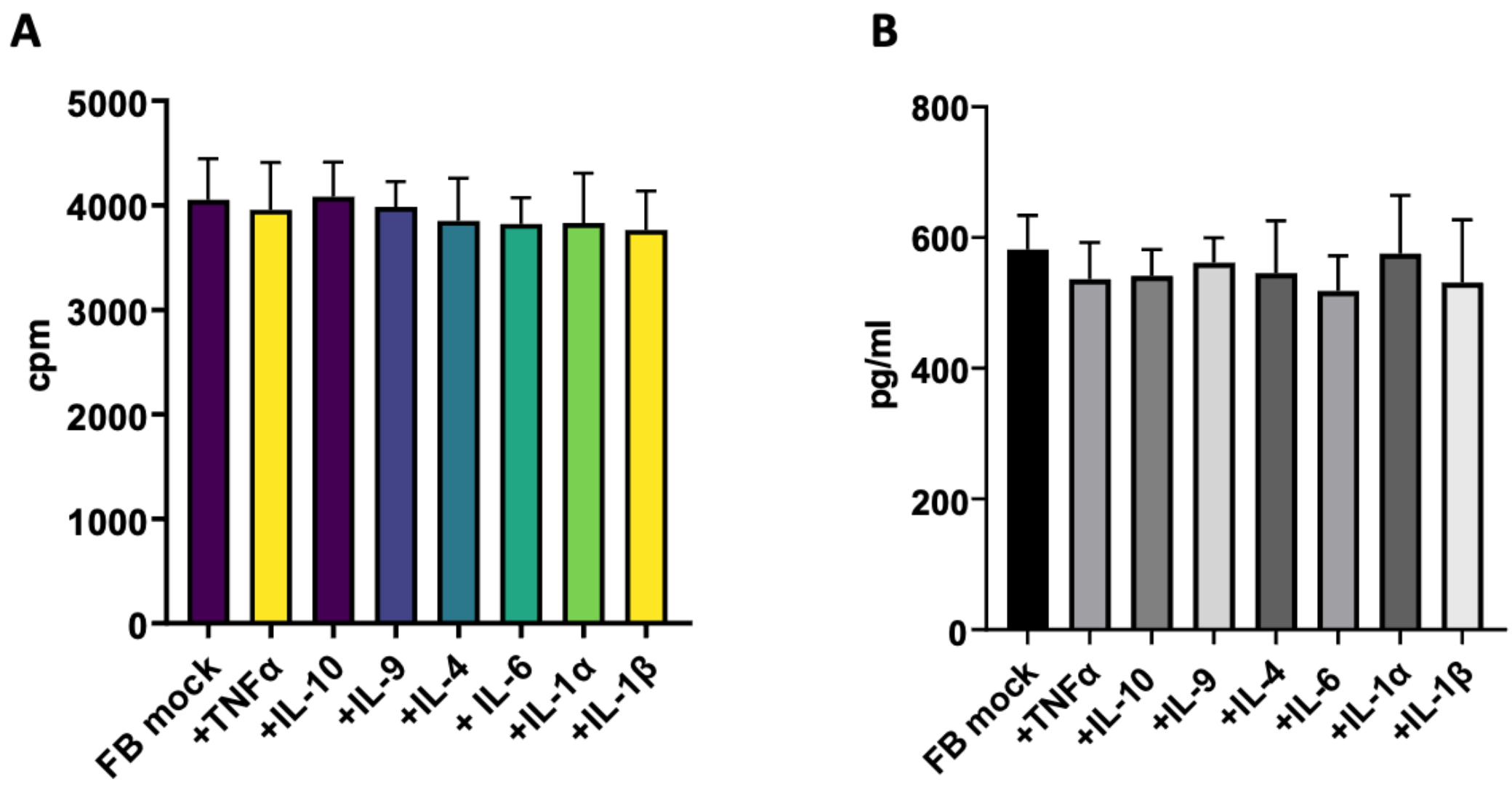

Supplementary Figure 5. Selected cytokines do not affect the proliferation of fibroblasts or collagen production. Fibroblasts (FBs) were stimulated with different cytokines, and their proliferative activity (**A**) and type I collagen production (**B**) were evaluated. Cell proliferation was assessed after 48 h of treatment by measuring [^{3}H]-thymidine incorporation, with results expressed as counts per minute (cpm). Type I collagen production was quantified by ELISA. Data are presented as mean ± SEM from four independent experiments.

Supplementary Figure 6

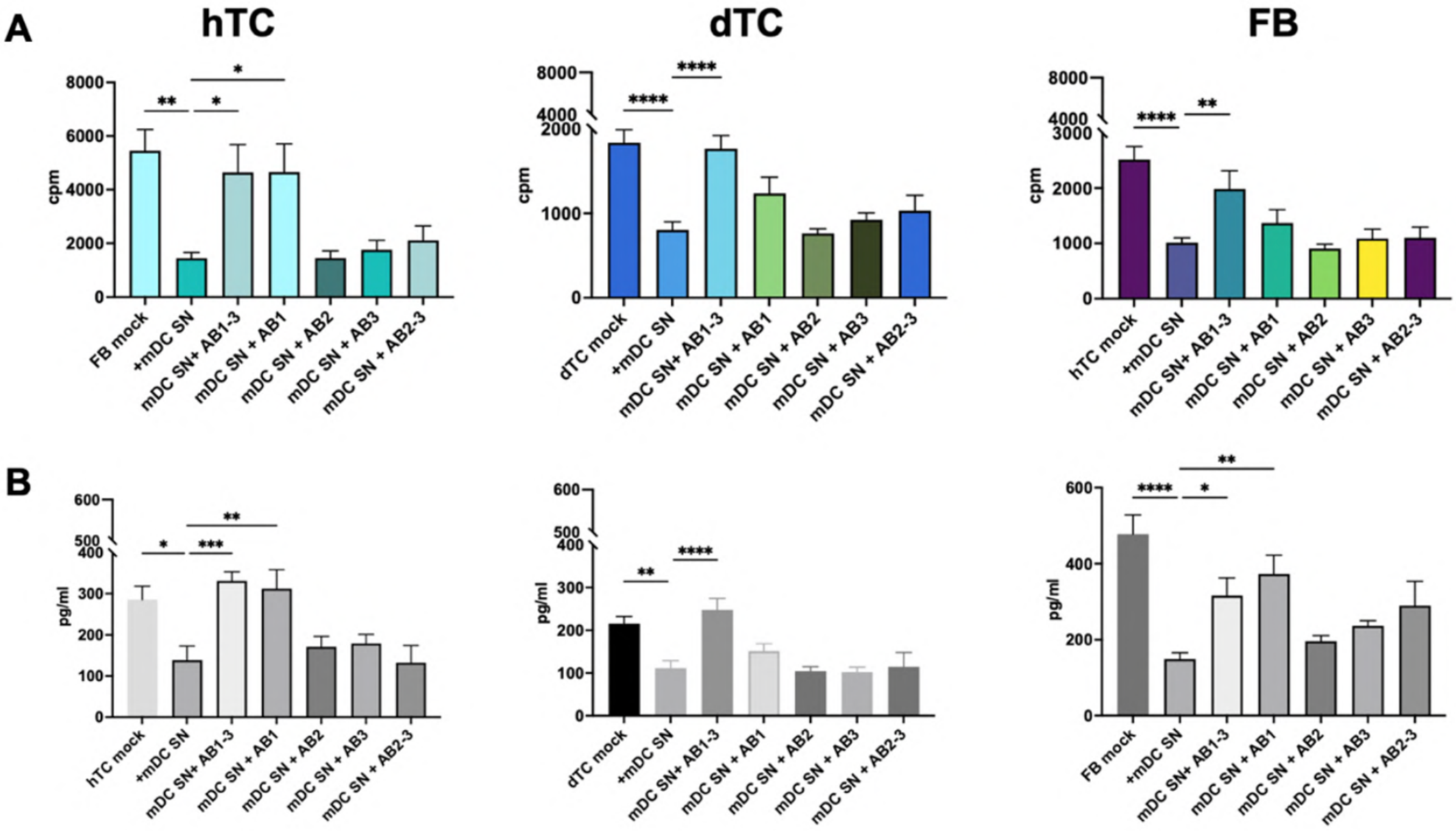

Supplementary Figure 6. Blocking of type I IFN recovered mDCs SN inhibitory effects. (A) Proliferation assay of TCs and FBs treated with supernatant from LPS-stimulated dendritic cells (mDCs) or left untreated (mock). Each condition was supplemented with blocking antibodies targeting type I IFN signaling, against the type I IFN receptor (AB1), IFN-α2 (AB2), and IFN-β (AB3). Proliferation was measured via thymidine incorporation after 48 hours. (B) Collagen production was evaluated using an ELISA assay. Cells were cultured for 2 days, after which the supernatant was collected, and type I collagen (COL1A1) levels were measured. Data represent the mean ± SEM from 3 independent experiments. Statistical analysis was conducted using a one-way ANOVA, and only significant differences are indicated: *$p < 0.05$, **$p < 0.01$, ***$p < 0.001$, ****$p < 0.0001$.

Supplementary Figure 7

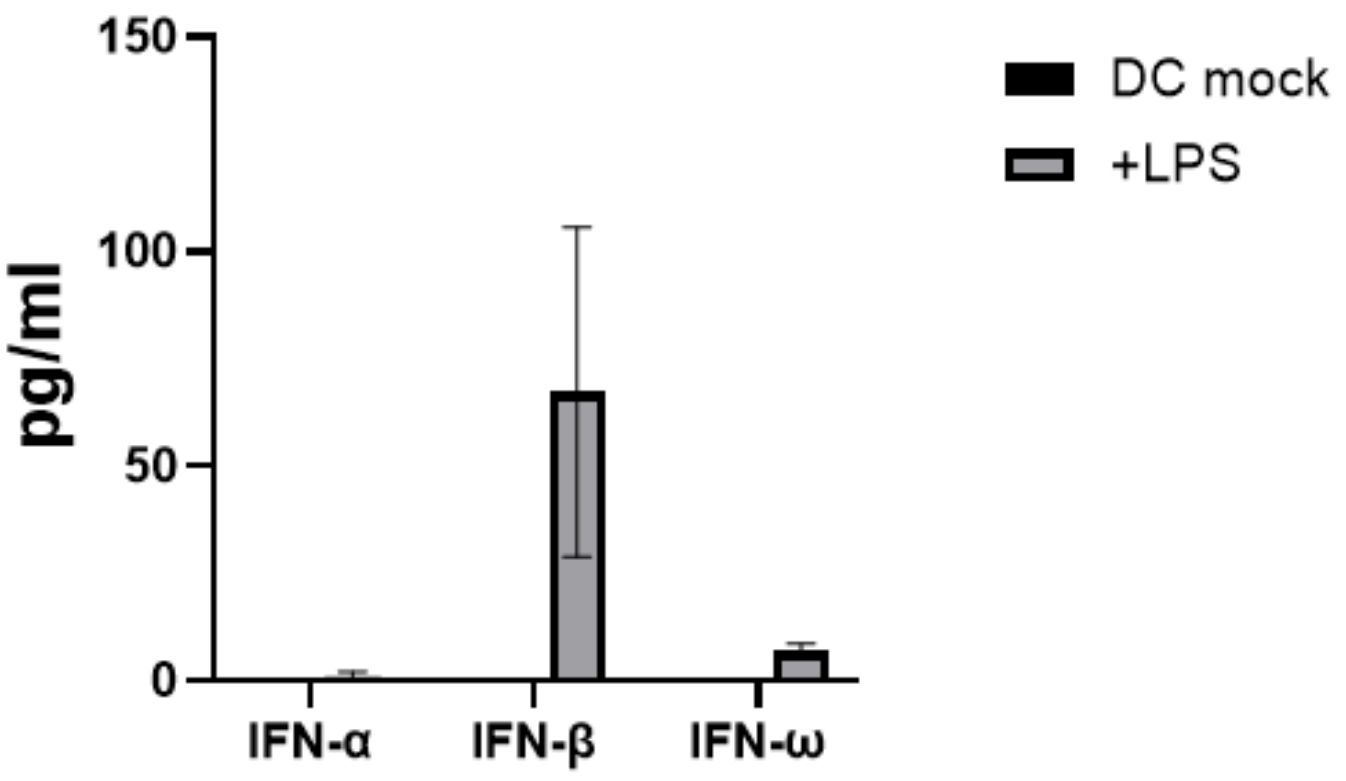

Supplementary Figure 7. Production and release of type I IFNs by LPS stimulated DCs. Immature DCs were stimulated with LPS (1 µg/mL) or were left untreated and cultivated for 2 days. Cell culture supernatants were harvested, and IFN-α, IFN-*β* and IFN-*ω* levels were measured via MILLIPLEX multiplex assays using the Luminex system. Data displays mean ± SEM of data from 3 independent experiments.
